# Dr. Hohnbaum's Observations on a Particular Species of Ophthalmic Disease, to Which Children of a Weak and Delicate Habit, and Particularly Those of the Lower Classes, Are Subject

**Published:** 1817-09

**Authors:** 


### *For the London Medical and Physical Journal.*

Dr. Hohnbaum's *Observations on a particular Species of Ophthalmic Disease, to which Children of a weak and delicate Habit, and particularly those of the lower Classes, are subject.* Communicated by Dr. von Embden.

THIS disorder is not always, though generally, combined with other marks of scrofula. It commences with sensibility to light, increasing gradually, so as to render it impossible to force the eyes open, as to expose more than a small portion of the eye-ball, the eye-lids closing spasmodically upon the fingers of the person attempting to open them, and the eye-ball involuntarily turning upwards. The conjunctiva appears somewhat reddened. Covering the eyes, and wrapping them up, generally does not suffice, on account of which the patients mostly lie on their faces, whilst the tears flow incessantly, and seem of so acrid a nature as to corrode the neighbouring parts. The first symptom of amendment is generally announced by a greater capacity of bearing the light. If the eye-lids continue closed for some time, maculæ frequently appear in the cornea, which, according to circumstances, are more or less considerable. Both eyes are constantly affected at the same time, and the disorder is apt to return till the patients arrive at a certain age. Change of weather, with the catarrhous affections resulting from it, appears to have a great influence on this disorder.

Dr. H. gives it as his opinion, that this morbid form does not properly deserve the name of ophthalmia; or at least that the inflammatory state is of the chronic kind, and the consequence of a morbid re-action of the lymphatic system upon the arterious. If the membranes of the eye are slightly inflamed, inflamed, it is in consequence of the acrid lachrymal fluid being retained. The febrile state prevailing in the beginning, the pain in the temples, and the good effect of the remedies, render it probable that the lachrymal gland is affected by the inflammation. The disorder is not so much to be considered an idiopathic affection as symptomatic of that kind of atrophy which arises from diseased glands, the relation of which to the lachrymal does not seem to depend on the nerves, but rather on the diminished and interrupted absorption of the abdominal glands, and an increased secretion in such regions as stand in a polar relation with them. An enormous accumulation of plastic matter, in consequence of the palsied state of the absorbents in those parts, may be, perhaps, the proximate cause.

Besides a proper regimen, such means as nature itself is often found to employ for removing accumulated lymph are to be employed. Of this kind are embrocations of unguent. lyttæ in the nape of the neck and behind the ears, in order to produce an artificial exanthemata,—mercurial purges of calomel and jalap; and, in order to limit the enormous lachrymal secretion, dry warmth is to be applied in the beginning, and afterwards warm fomentations, with extractum opii aquosum, super-acetate of lead, and spirit. anthos.

---

We have interfered as little as possible with the pathological theories of this paper, as they are intermixed with, and make part of, the description.—EDIT.

